# Prevalence study on self-declared work accidents in areas covered by family health strategies: a cross-sectional study

**DOI:** 10.1590/1516-3180.2019.0325.R1.06112019

**Published:** 2020-04-22

**Authors:** Karen Thalita Pereira, Ana Carolina Rodrigues de Sá Silva, Luiz Felipe Silva

**Affiliations:** I MSc. Doctoral Student and Nurse, Universidade Estadual Paulista (UNESP), Guaratinguetá (SP), Brazil.; II MSc. Doctoral Student and Environmental Engineer, Universidade Estadual Paulista (UNESP), Guaratinguetá (SP), Brazil.; III PhD. Mechanical Engineer and Associate Professor, Institute of Natural Resources, Universidade Federal de Itajubá (UNIFEI), Itajubá (MG), Brazil.

**Keywords:** Accidents, occupational, Occupational health, Accidents, Public health, Accident prevention, Accident at work, Underreporting, Epidemiological characteristics

## Abstract

**BACKGROUND:**

Occupational accidents are a complex phenomenon and a major public health problem. Occupational health surveillance actions are essential for prevention of injuries of this nature.

**OBJECTIVE:**

To ascertain the prevalence of and the variables associated with occupational accidents in the city of Itajubá (MG).

**DESIGN AND SETTING:**

A cross-sectional study with a quantitative approach, based on a household survey with random sampling, was conducted in areas covered by the Family Health Strategy (FHS) in Itajubá (MG).

**METHODS:**

Questionnaires were applied to 292 people*.* The data were analyzed by means of logistic regression.

**RESULTS:**

The prevalence of occupational accidents was 8.6%. The underreporting rate was 60.0%. The scenario for these accidents, according to the model established through the regression analysis, was most likely to involve males who declared their skin color as white and who did not have a formal employment contract.

**CONCLUSION:**

This study makes a contribution towards unveiling the relationship between healthcare and work, and thus serve as support for the development of strategies to prevent underreporting. Lastly, the results provide the basis for future public health intervention actions and for future studies.

## INTRODUCTION

Occupational accidents are a complex phenomenon and a major public health problem that requires attention from all sectors, including healthcare, industry or services, because these accidents generate high socioeconomic costs.^1^

Work that is carried out under unsuitable conditions can cause various adverse consequences for humans, both physically and psychologically. These consequences may give rise to impairments that lead to absenteeism, premature retirement, reduced income, job disruption for the family, temporary incapacitation, physical or psychological pain, mutilation and even death. Faced with this reality, it is essential that this topic is discussed in order to conduct studies that show the unfavorable impacts of work on workers’ health.^2^^,^^3^ One of the main causes of health problems among workers is the fragility of the health and safety structure, along with inadequate risk management.^4^

In order to implement care, surveillance, preventive actions and health promotion actions, the National Network for Integral Attention to Worker’s Health (RENAST)^5^ was created in Brazil in 2002. This is a complex network that includes production and management of knowledge and actions to be developed for workers’ health. The network is composed of state and regional reference centers for occupational health (CERESTs) that have the objective of acting towards health promotion, preventive actions, surveillance, assistance and rehabilitation relating to workers’ health in both urban and rural settings, irrespective of employment relationships and the type of status in the labor market.^6^

All work accidents must be reported in the notifiable hazards information system (Sistema de Informação de Agravos de Notificação, SINAN). This system has been implemented gradually since 1993 and is mainly fed by notifications and investigations of cases of diseases and injuries that are listed as matters for compulsory notification. When used effectively, this system makes it possible to identify the epidemiological reality of a given geographical area.^7^

In the case of accidents among workers who are insured through social security, it is necessary to open a work accident notice (Comunicação de Acidente de Trabalho, CAT). The aim of this is to inform the social security system about the occurrence of an accident at work, even if this event does not lead to a worker’s absence.^8^

Because health problems among workers can give rise to a variety of types of harm and consequences, it is necessary to identify the associated factors. The official statistics on occupational accidents are insufficient to delineate such problems in an in-depth and accurate manner that allows the realities to be understood. It is important to note that underreporting occurs, especially when it comes to workers who are not covered by social security.^9^ Most of the time, the reliability of information among government agencies is compromised because of the large numbers of informal workers with no employment relationship.^3^

Occupational health surveillance actions are essential for prevention of injuries of this nature. Persistent underreporting of occupational accidents, which is a public health problem, constitutes a significant obstacle to the effectiveness of these measures. It also has a harmful and important repercussion on labor and social security rights.^10^

## OBJECTIVE

To ascertain the prevalence of and the variables associated with occupational accidents in the city of Itajubá (MG), Brazil.

## METHODS

### Place of study

The study was conducted in the municipality of Itajubá, which has a population of 90,658 inhabitants and is located in the southern region of Minas Gerais.^11^ The industrial district of Itajubá is considered one of the largest in the south of Minas Gerais, with large and medium-sized industries, in the fields of auto parts, military equipment, helicopters and soaps, among others, generating approximately 2,500 jobs.

### Study population

The study population comprised the population that is covered by the Family Health Strategy (FHS), which covers approximately 41.33% of the inhabitants of the municipality, including both the rural and the urban area, with 13 FHS units located in the urban area and two FHS units in the rural area.^12^

### Study design

This was a cross-sectional quantitative study based on a household survey.

### Sample definition and data collection

The sample size was determined based on the method proposed by Gil.^13^ Through this method, it was defined as 265 subjects. A safety margin of 10% was also taken into consideration in relation to the sample size, thus resulting in a total of 292 homes to be visited.

The data collection was based on the method used by Cordeiro et al.,^10^ in which the steps were the following:

- Preliminary survey of FHS data to identify whether homes were occupied or unoccupied;
•Sample definition by means of a simple random process;
- Data collection.

Through the data collection, which was performed through application of individual questionnaires in September 2015, it was sought to reveal the prevalence and underreporting of accidents. Closed homes, i.e. those that were classified as occupied, but with no residents present at the time of the visit, were visited again on up to two more occasions, on different days and at different times. If, on the third visit, the home was still closed, it was discarded from the sample and replaced. Among the homes visited, if the resident (aged eighteen years or over) refused to participate, this home was discarded from the sample and replaced.

Subjects who agreed to respond to the questionnaire were asked whether any of the residents of their household over the age of sixteen years had suffered any accident of any kind (work, traffic, domestic, etc.) in the last two years. If so, a return to the address was scheduled, to interview this individual and confirm the occurrence of the alleged accident. When its occurrence was confirmed, a semi-structured questionnaire was applied to the subject involved in the event.

A pre-test was performed using 20 subjects in different homes in order to ascertain the adequacy of the questionnaire and to validate. The questionnaire had been drawn up by the authors of the present study. This process was undertaken to evaluate the clarity of the questions and avoid redundancy among them, and to assess the effectiveness of the questionnaire.

The questionnaires were applied by the authors themselves, with the support of a duly trained research auxiliary. The aim was to identify work accidents that occurred during the period from August 2013 to August 2015.

The criteria for subjects to be included in the study were that they needed to have lived in Itajubá for at least one year and to have had occupational activity in Itajubá for at least one year; and that their homes were covered by the FHS. The criteria for them to be excluded were that they had lived in Itajubá for less than one year or carried out occupational activities in another municipality; and that their homes were not covered by the FHS.

For the respondents who were paying social security contributions and had suffered an occupational accident, the proportion of underreporting was calculated as the ratio between the observed number of non-notified work accidents that should have been subject to compulsory notification and the total number of events (reported and not reported).

### Data analysis

The associations between the dependent variable (work accidents suffered during the two-year period) and the explanatory variables were studied through multiple non-conditional logistic regression, to make it possible to control for confounding variables, in order to avoid possible interferences regarding the outcome of the study.

The variables for which there were more than two response options were dichotomized at the time of their insertion in the EPI-INFO software, version 3.5.1TM (2008). Thus, marital status was separated into married/stable partnership versus single/separated; skin color into black versus white/brown; and employment status into registered versus unregistered/self-employed.

### Ethical issues

The present study followed all the ethical principles, and data collection was only done after approval had been obtained from the Research Ethics Committee of the Medical School of Itajubá (MG), under the constituted opinion number 1,111,978 (approval date: June 17, 2015). Resolution 196/96 and the principles of autonomy and privacy were respected. People who agreed to participate in the study signed an informed consent statement.

## RESULTS

Among the questionnaires applied, 292 were considered valid. The sociodemographic data of these 292 study participants, whose mean age was 45.1 ± 16.8 years, and the prevalence of underreporting are shown in Table 1.

**Table 1.** Distribution of the sociodemographic data of the 292 interviewees and of the prevalence of work accidents according to stratum, in Itajubá (MG) between August 2013 and August 2015

Explanatory variable	n (%)	Prevalence of work accidents by stratum n (%)
Skin color		
White	168 (57.7)	20 (11.9)
Brown	114 (39.0)	4 (3.5)
Black	10 (3.4)	1 (10.0)
Marital status		
Single	88 (30.1)	5 (5.7)
Married	178 (61.0)	16 (9.0)
Separated	7 (2.3)	1 (14.3)
Stable partnership	3 (1.0)	3 (100.0)
Widower	16 (5.5)	0 (0.0)
Schooling		
Elementary	135 (46.2)	12 (8.9)
High School	144 (49.4)	13 (9.0)
Higher education	6 (2.1)	0 (0.0)
Illiterate	7 (2.3)	0 (0.0)
Location of home		
Urban area	256 (87.8)	24 (9.4)
Employment relationship		
Working with formal employment contract	104 (35.6)	13 (12.5)
No employment contract	52 (17.9)	8 (15.4)
Self employed	21 (7.3)	4 (19.0)
Retired	50 (17.1)	0 (0.0)
Unemployed	65 (22.1)	0 (0.0)
Gender		
Male	118 (40.5)	20 (6.8)
Underreporting of work accidents	60.0%	
Total	292 (100)	25 (8.6)

There were 25 workers who had suffered industrial accidents, and the distribution of their characteristics is shown in Table 2. The predominant characteristic of these individuals who sustained injuries during the observation period was that they were married white men who had not been trained to perform the function.

**Table 2.** Distribution of the sociodemographic characteristics of the 25 workers who suffered an accident at work, Itajubá (MG), 2013-2015

Explanatory variable	n (%)
Not trained	18 (72.0)
Medical care	
Hospital	17 (68.0)
Outpatient	1 (4.0)
In the company	6 (24.0)
Primary healthcare unit	1 (4.0)
Length of time in the profession in years [mean (standard deviation)]	8.9 (8.7)
Hours elapsed from the beginning of work to the time of the accident [mean (standard deviation)]	4.9 (2.9)
With remission from work	15 (60.0)
Days of absence [mean (standard deviation)]	42.9 (96.2)
Place of work accident	
Industry (factories and machine shops)	8 (32.0)
Construction	8 (32.0)
Butcher’s shop	3 (12.0)
Restaurant	2 (8.0)
Family home	1 (4.0)
Laboratory	1 (4.0)
School	1 (4.0)

Among all the 25 workers who suffered an accident at work, 12 were not registered or were self-employed, and five of these were paying social security contributions but did not notify SINAN. The other 13 workers had formal employment contracts and 10 did not open a CAT after their accident. Thus, in total, 15 workers among these 25 accident victims did not open a CAT or make the mandatory notification to SINAN, thereby representing an underreporting rate of 60.0%.

### Logistic regression analysis

The model that was considered to present the most appropriate fit was one that considered the accidents that occurred over the entire period of the study.

Table 3 shows the results obtained by means of the univariate and multiple analysis, which shows the respective prevalence odds ratio, 95% confidence interval (CI) and P-value of the explanatory variables.

**Table 3.** Univariate and multiple analyses presenting odds ratios, 95% confidence intervals and P-values for the dependent variable “work accidents over the two-year period”, Itajubá (MG), 2013-2015

Univariate analysis			
Explanatory variables	OR*	95% CI	P-value		
Skin color (white)	3,13	1.15-8.52	0.015		
Marital status (married)	1.14	0.49-2.66	0.75		
Schooling (high school)	0.97	0.43-2.19	0.95		
Location of home (urban area)	3.51	0.46-26.54	0.14		
Formal employment contract	0.51	0.33-0.77	< 0.001		
Gender (male)	6.59	2.42-17.96	< 0.001		
Age	0.97	0.95-10.02	0.06		
Multiple analysis			
Explanatory variables	OR**	95% CI	Coefficient (β)	P-value
Gender (male)¹	6.28	2.25-17.50	1.84	0.0005
Formal employment contract¹	0.05	0.03-0.08	- 0.07	0.0024
Skin color (white)¹	3.63	1.28-10.30	1.29	0.0152

OR = odds ratio; CI = confidence interval.

¹The variables that were entered in the adjustment; *Crude odds ratio; **Adjusted odds ratio.

## DISCUSSION

The prevalence of occupational accidents over the two-year period from August 2013 to August 2015 was 8.6%: 3.5% in the first year and 5.1% in the second year. The annual values found are close to those reported from the study developed by Binder et al..^14^ On the other hand, Cordeiro et al.^10^ found that the prevalence of typical work accidents over a three-month period was 0.7%. It is important to mention that these two studies, which included the whole municipality, used different methodologies regarding the definition of sampling. Differently, the present study had the particular feature of only involving people who were covered by the FHS.

Among the 25 workers who suffered work accidents, 15 workers (10 with employment registration and five without registration or self-employed) did not open a CAT or give notification through SINAN. This figure is equivalent to a prevalence of underreporting of 60.0%, which is a very worrying proportion, since it shows that more than half of the work-related accidents were not reported. Of this proportion, 55.6% belong to the category of workers with registration and 27.7% to workers without registration or who were self-employed who were nonetheless paying contributions to the National Social Security Institute. Among the five workers who were making social security contributions but who were unregistered or self-employed, none of them made a notification of the accident.

Underreporting compromises public policies for promoting workers’ health. Among the main reasons for underreporting are an understanding that the event was not of sufficient severity,^15^ a duration of sick leave of less than sixteen consecutive days, for which there is no concession of benefits,^16^ occurrence of accidents among informal workers,^17^ poor functioning of health surveillance for workers and weak capacity and responsibility among municipalities for adequate identification and notification of cases.^18^

The National Health System (Sistema Único de Saúde, SUS), through the FHS, has a very important role in identifying underreporting of occupational accidents, since primary care is the gateway for people to access healthcare services. Hence, it needs to be possible for the entire community covered to be received within primary care, so that links between patients and professionals can be established. Community health agents work side-by-side with the community and so are able to be better informed about whether occupational accidents have occurred or not. In addition, a relationship of trust is established through coexistence between these health agents and patients. Consequently, health agents can obtain a greater amount of information regarding what is happening with regard to the health of members of the community who live in the homes that are covered.

Another way to improve surveillance relating to occurrences of occupational accidents is to implement additional CERESTs. These centers are specialized places that have a responsibility for hosting these workers, thereby clarifying their doubts and acting towards promotion, protection and recovery of workers’ health.

Regarding the prevalence of disability due to work accidents, a proportion of 60% was found, similar to what was found in another study.^19^

As observed in the present study, Rios et al.^15^ also found a greater number of work accidents among men. However, another study^20^ found that these accidents were predominantly among women. This finding can be explained by the fact that the latter study was carried out among healthcare professionals, among whom women predominate.

The average age of the victims was 38.7 years (standard deviation, SD = 10.05), as has also been found in other studies,^15^^,^^20^^,^^21^^,^^22^ in which mean ages of between 33 and 39 years were found.

Regarding skin color, white skin color prevailed in the present study. This result differed from what was found by Santana et al.,^23^ who observed higher prevalence of accidents involving black-skinned people in Salvador, Bahia. The explanation for this difference is that the city of Itajubá has a predominantly white population, while the region of Salvador has a population of black and brown skin colors.^24^

Regarding schooling, Santana et al.^23^ observed that the prevalence was 72% among subjects with less than high school education. This differed from what was found in the present study, in which the proportion was 48%.

Regarding medical care, most of the patients were attended to in the emergency room, unlike in another study,^23^ in which the majority received care in outpatient clinics. This may indicate the possibility that the work accidents in Itajubá were more serious.

Most of the accident victims were treated within the public healthcare network, as was also found in another study.^10^ The public healthcare network can act as an essential support for strengthening the information system on occupational accidents.^10^ In addition, in line with these measures, CERESTs that are properly organized and structured have an important role in strategies contributing towards notification of occupational accidents in all municipalities.^25^ Demonstrating The importance of implementing a CEREST in the place of the present study was thus demonstrated, with the aim of reducing the level of underreporting.

In the current survey, 32% of the accidents occurred in environments and processes relating to industrial activity, and another 32% in construction. In a study by Binder et al.,^14^ the secondary sector (i.e. industrial activity) accounted for 42.1% of accidents, while construction accounted for 18.4%. In the present study, working as a butcher took third place regarding the prevalence of accidents. Rios et al.^15^ found a strong association between occurrences of accidents and working as a butcher, which can be explained in terms of the risks present in the work process and poor management of these risks.

The main concern regarding occupational accidents is that many of the workers who suffer these accidents do not have any formal employment contract (46%). Thus, they are sometimes left homeless and without income when these accidents occur.^26^^,^^27^

Regarding the variable of age, although this was an important biological factor, it was decided not to place it in the multiple analysis, so that the significance of the model would not be reduced.

Through logistic regression, it was possible to observe that male workers were more likely to suffer work accidents, as had already been observed in the literature.^21^^,^^22^^,^^23^^,^^28^^,^^29^^,^^30^^,^^31^^,^^32^

Regarding the situation of being registered or not, it was observed that registration acted as a protection factor in relation to work accidents. No association was found between these two factors (registration and work accidents) in the literature. Therefore, this possible association deserves to be better addressed in future research, with the hypothesis that there is a relationship between work accidents and informal work. The idea would be that when worker are registered, this presupposes there is greater concern on the part of the employer about adoption of preventive measures. It should also be noted that outsourced workers are registered. Outsourcing is another important variable that has not been addressed, and this should be considered in future research on the genesis of occurrences of accidents.

White skin color was also a significant factor in increasing the likelihood of occurrences of accidents, as had previously been observed in other studies.^33^^,^^34^

Lastly, as limitations of the present study, it can be noted that it had the typical limitations of a cross-sectional study, such as information bias and memory bias, since information about workers who have suffered accidents at work needs to be validated through medical records or diagnoses. The present study depended exclusively on the memories of the people who were interviewed.

## CONCLUSION

Our study found that the prevalence of occupational accidents in the city of Itajubá (MG) was close to what had been seen in the literature. In relation to the variables associated with occupational accidents, the most favorable scenario for occurrences of a work-related accident, found through logistic regression, consisted of situations in which work was done without a formal contract, by males and by people with white skin color. Having a formal employment contract acted as a protection factor.

It is recommended that further studies should be conducted, with exploration of other variables, such as outsourcing, the relationship between work accidents and lack of a signed employment contract, among others that may help in explaining the work accident phenomenon.
